# Genetic Diversity of Rift Valley Fever Strains Circulating in Namibia in 2010 and 2011

**DOI:** 10.3390/v12121453

**Published:** 2020-12-16

**Authors:** Gian Mario Cosseddu, Kudakwashe Magwedere, Umberto Molini, Chiara Pinoni, Sigfried Khaiseb, Massimo Scacchia, Maurilia Marcacci, Andrea Capobianco Dondona, Fabrizia Valleriani, Andrea Polci, Federica Monaco

**Affiliations:** 1 Istituto Zooprofilattico Sperimentale dell’Abruzzo e del Molise “G. Caporale”—IZSAM, Campo Boario, 64100 Teramo, Italy; c.pinoni@izs.it (C.P.); m.scacchia@izs.it (M.S.); m.marcacci@izs.it (M.M.); andrea@farm4trade.com (A.C.D.); f.valleriani@izs.it (F.V.); a.polci@izs.it (A.P.); f.monaco@izs.it (F.M.); 2 Central Veterinary Laboratory (CVL), 13187 Windhoek, Namibia; gwedas@yahoo.co.uk (K.M.); u.molini76@gmail.com (U.M.); khaisebs@gmail.com (S.K.)

**Keywords:** Rift Valley fever, Namibia, full genome sequence, phylogenetic analysis

## Abstract

Outbreaks of Rift Valley fever (RVF) occurred in Namibia in 2010 and 2011. Complete genome characterization was obtained from virus isolates collected during disease outbreaks in southern Namibia in 2010 and from wildlife in Etosha National Park in 2011, close to the area where RVF outbreaks occurred in domestic livestock. The virus strains were sequenced using Sanger sequencing (Namibia_2010) or next generation sequencing (Namibia_2011). A sequence-independent, single-primer amplification (SISPA) protocol was used in combination with the Illumina Next 500 sequencer. Phylogenetic analysis of the sequences of the small (S), medium (M), and large (L) genome segments of RVF virus (RVFV) provided evidence that two distinct RVFV strains circulated in the country. The strain collected in Namibia in 2010 is genetically similar to RVFV strains circulating in South Africa in 2009 and 2010, confirming that the outbreaks reported in the southern part of Namibia in 2010 were caused by possible dissemination of the infection from South Africa. Isolates collected in 2011 were close to RVFV isolates from 2010 collected in humans in Sudan and which belong to the large lineage containing RVFV strains that caused an outbreak in 2006–2008 in eastern Africa. This investigation showed that the RVFV strains circulating in Namibia in 2010 and 2011 were from two different introductions and that RVFV has the ability to move across regions. This supports the need for risk-based surveillance and monitoring.

## 1. Introduction

Rift Valley fever is a severe disease of livestock, causing abortions and neonatal mortality in domestic ruminants. The disease is caused by RVFV, an enveloped RNA virus of the *Phlebovirus* genus (Family *Phenuiviridae*). The infection with RVFV can cause mild to severe illness in humans [1]. The disease transmission occurs primarily by mosquitoes, which are vectors and reservoirs of the virus [2,3,4]. Close contact with infected tissues or body fluids might cause infection in humans [5].

The virus was first identified in the Great Rift Valley in Kenya in the 1930s [6,7]. Although RVFV isolation and serological evidence was reported in greater parts of the African continent, the disease is considered endemic to eastern and southern Africa (Sudan, Somalia, Kenya, Zimbabwe, Zambia, Mozambique, Namibia, Madagascar, Tanzania, and South Africa) and in the western countries of Mauritania, Senegal, and Niger. Substantial outbreaks have also been reported in Egypt, Saudi Arabia, and Yemen [8,9], amongst others, in the past, thus demonstrating the potential for further spread of the pathogen into disease-free regions [10,11].

RVF was frequently reported in South Africa from 1950 to 2011. Three major epidemics affected large areas of the country: (i) from December 1950 until April 1951 (season 1950–1951), this was the first time RVF had been diagnosed in South Africa; (ii) the largest RVF epidemic began in 1973 and continued until 1976 for three consecutive seasons (1973–1974, 1974–1975, 1975–1976); (iii) during the first half of 2010, a major epidemic occurred (season 2009–2010), followed by a smaller one during 2011 (season 2010–2011) [11]. In 2010, South African veterinary authorities reported more than four hundred RVF outbreaks to the World Organization for Animal Health (OIE), with tens of thousands of deaths in susceptible livestock [12]. In January 2010, RVF outbreaks were reported in the eastern Free State Province. The disease spread progressively to the Northern Cape Province close to the border with Namibia.

In Namibia, antemortem inspection (AMI) aims to protect public health by minimizing hazardous material that enters in the food chain through routine animal health surveillance targeted for the detection and monitoring of animal diseases and welfare problems. During routine AMI, a farmer supplied sheep to an abattoir in May 2010, and upon arrival, seven sheep were dead on arrival at the abattoir, and ten sheep were moribund. At postmortem inspection, the sheep from the same farm had cyanotic tongue and pneumonia consistent with findings usually reported in cases of blue tongue virus infections; however, a few sheep had mild liver congestion, turning mild bronze to yellow in color. Only one healthy ewe from a different farm in a large consignment presented signs of a recent abortion. The suspicion for RVF and its inclusion as a differential diagnosis was made based on the outbreak of RVF that was ongoing in the Republic of South Africa, the close proximity of the farm to the RSA border, some suspicious postmortem lesions observed in the liver, and the single healthy ewe that had visible signs of abortion. Organ samples from the dead animals as well as blood samples from sick and health animals were send to the Central Veterinary Laboratory (CVL) in Windhoek for confirmation of blue tongue virus and RVF by the real time reverse transcription polymerase chain reaction (RT-PCR) technique.

Subsequent to the confirmation of RVF and intensified active surveillance on farms, seven outbreaks were reported in different geographic locations in sheep holdings in the southern Namibian regions of Hardap and Karas. The presence of infection was confirmed by RT-PCR in clinically suspected flocks [13]. The distribution of suspicious cases in the southern part of Namibia did not infer any particular epidemiological pattern consistent with arboviral infections, where cases of RVF most commonly occur after periods of heavy rainfall, which lead to an abundance of mosquitoes, coupled with the traceable movements of hosts, physical resources, and vectors between farms, which causes the geographical transfer of the RVFV. Furthermore, the disease did not appear to present the typical clinical picture of classical RVF, where one would have expected mass abortions, mass lamb mortalities, and more moribund and sick animals. Preliminary investigations indicated some degree of vaccination for RFV, which might have explained the lack of typical signs of RVF, coupled with the decrease of mosquito activity due to the onset of colder weather in the affected regions. Historically, RVFV lineage H, which was responsible for the 2010–2011 South African human epizootics, contained an antecedent from Namibia in 2004 [14].

In May 2011, three outbreaks were reported in livestock in the Oshikoto region, northeast of Etosha National Park [15]. Sampling of wild ruminants in Etosha National Park resulted in the detection of viremic springboks (*Antidorcas marsupialis*), wildebeest (*Connochaetes taurinus*), and black-faced impala (*Aepyceros melampus petersi*) by RT-PCR and serology [16]. The areas where the RVFV strains were detected are indicated in Figure S1.

The genome of RVFV is composed of three single-stranded segments of negative-sense RNA, of around 12 kb in total. The L (large) segment encodes for the viral RNA polymerase. The M (medium) segment encodes for the G1 and G2 membrane glycoproteins and for two non-structural (NS) proteins NSm1 and NSm2 of 78 kDa and 14 kDa, respectively. The S (small) segment codes for the nucleocapsid protein N and for the non-structural NSs [17,18,19]. The RVFV genome is characterized by low genetic diversity (between 1 and 5%) and by the existence of a single RVFV serotype. Alternating replication between mammalian and arthropod hosts may constrain the evolution of the virus genome, limiting the genetic diversity of the circulating field strains [20,21]. The segmented structure of the RVFV genome makes the occurrence of recombination events through reassortment possible [8,22].

This study describes whole-genome sequencing and phylogenetic analysis of the RVFV strains isolated in Namibia during 2010 and 2011.

## 2. Materials and Methods

### 2.1. Virus Strains

The RVFV isolate, obtained in 2010, was from an infected sheep sampled during a disease outbreak that occurred in May–June 2010 in Namibia. Virus isolation was conducted at the Istituto Zooprofilattico Sperimentale dell’Abruzzo e del Molise in Teramo, Italy, by infecting Vero E6 cells (ATCC CRL-1586 VERO C1008) with the RT-PCR positive uterus sample homogenated (10% weight/volume) in phosphate buffered saline (PBS) [13]. Samples from 2011 were from clinically healthy springbok sampled in May–June 2011 in Etosha National Park. Sequencing was attempted from the serum.

### 2.2. Sequencing

In this study, we carried out full genome sequencing of two RVFV strains collected in Namibia in 2010 and 2011. The sequence of the Namibia_2010 strain was generated by amplifying overlapping sections of the genome. RT-PCR primers that were used are shown in Table S1. Amplicons used for direct sequencing were purified with the Qiaquick PCR purification kit (Qiagen, Venlo, The Netherlands). The reaction was carried out using the Big Dye Terminator kit (Thermo Fisher Scientific, Waltham, MA, USA). Nucleotide sequences were obtained using the DNA sequencer ABI PRISM 3100 (Thermo Fisher Scientific). Amplification and sequencing of each PCR fragment were repeated twice. Sequenced raw data were assembled using Contig Express Vector NTI suite 9.1 (Thermo Fisher Scientific).

Namibia_2011 were sequenced by using the sequence-independent, single-primer amplification (SISPA) technique according to the protocol of Allander et al. [23] in combination with the Illumina Next 500 platform (Illumina, San Diego, CA, USA, https://www.illumina.com). Raw reads after cleaning were de novo assembled using SPADES [24]. Returned contigs were checked for artefacts and compared to the GenBank database. Contigs showing similarity with host genomes or bacterial sequences were removed. Contigs accounting for a partial but reliable assembly were assigned to RVFV.

The sequences generated were submitted to GenBank under accession numbers MT561459, MT561460, MT561461, MT561462, MT561463, and MT561464.

### 2.3. Phylogenetic Analysis

**Figure 1:**
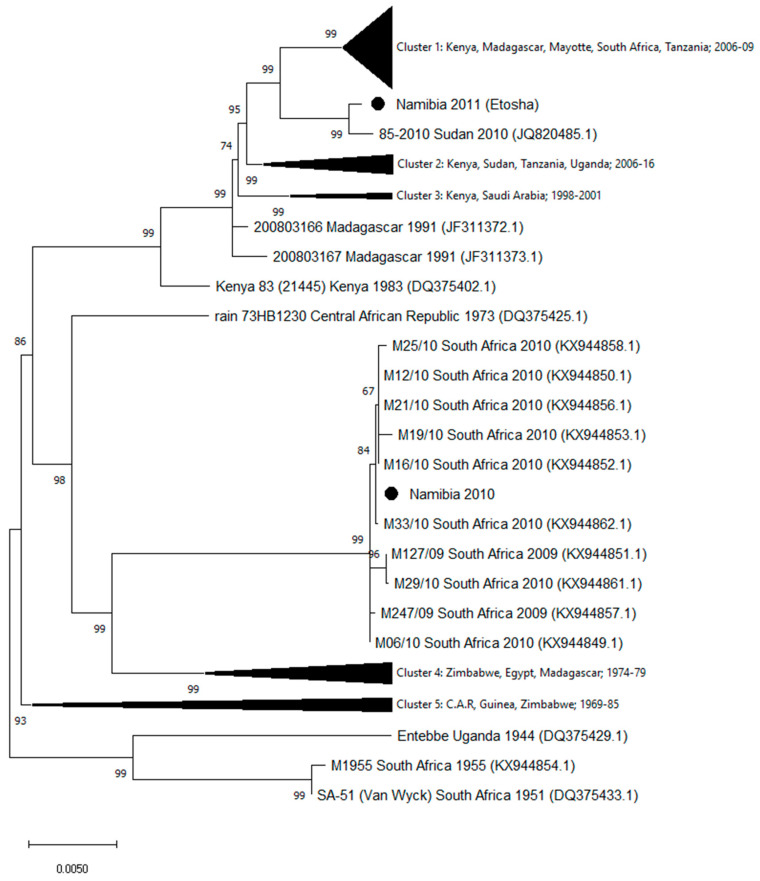
Phylogenetic tree of Rift Valley fever virus (RVFV) generated by the maximum likelihood method with bootstrap (500 replicates) based on 6404 nucleotides of the L genomic segment of RVFV. Analysis involved 94 nucleotide sequences available in GenBank. Compressed Cluster 1 includes 43 sequences from Kenya, Madagascar, Mayotte, South Africa, and Tanzania, collected in the period 2006–2009; Cluster 2: ten sequences from Kenya, Sudan, Tanzania, and Uganda collected in 2006–2016; Cluster 3: three sequences from Kenya and Saudi Arabia, collected in the period 1998–2001; Cluster 4: eleven sequences from Zimbabwe, Egypt, and Madagascar, collected in 1974–1979; Cluster 5: seven sequences from C.A.R, Guinea, and Zimbabwe, collected in 1969–1985. The strain names and accession numbers of the sequences included in the compressed clusters are listed in Table S2.

**Figure 2:**
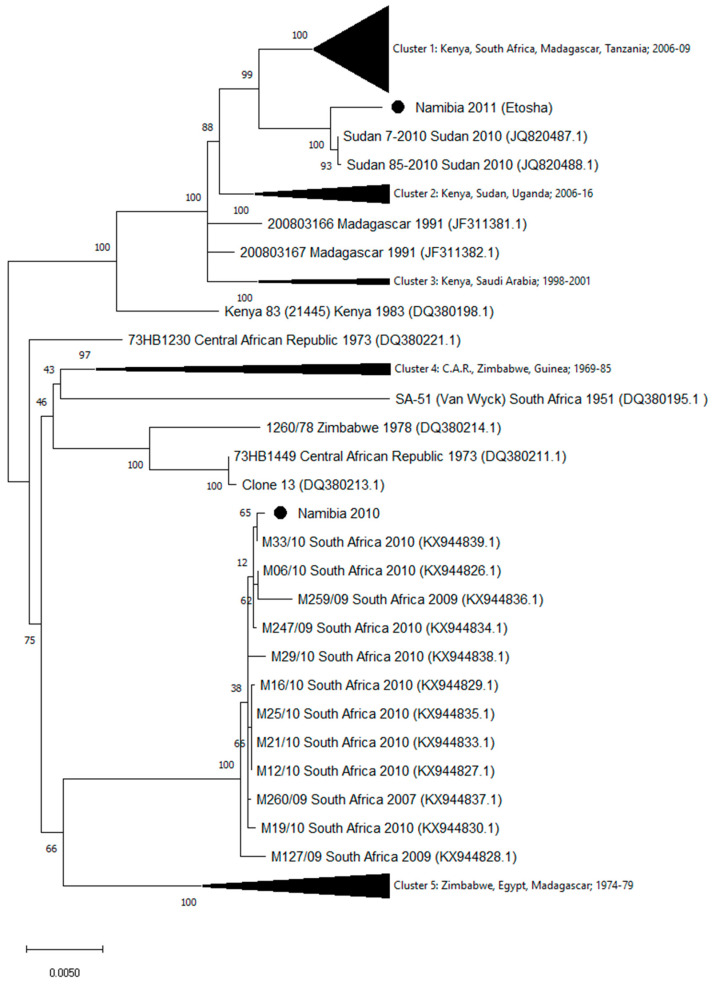
Phylogenetic tree of RVFV generated by the maximum likelihood method with bootstrap (500 replicates) based on 3885 nucleotides of the M genomic segment of RVFV. Analysis involved 103 nucleotide sequences available in GenBank. The compressed Cluster 1 includes 47 sequences from Kenya, South Africa, Madagascar, and Tanzania, collected in the period 2006–2009; Cluster 2: ten sequences from Kenya, Sudan, and Uganda, collected in 2006–2016; Cluster 3: three sequences from Kenya and Saudi Arabia collected in 1998–2001; Cluster 4: six sequences from C.A.R., Zimbabwe, and Guinea, collected in 1969–1985; Cluster 5: thirteen sequences from Zimbabwe, Egypt, and Madagascar, collected in 1974–1979. Strain names and accession numbers of the sequences included in the compressed clusters are listed in Table S3.

**Figure 3:**
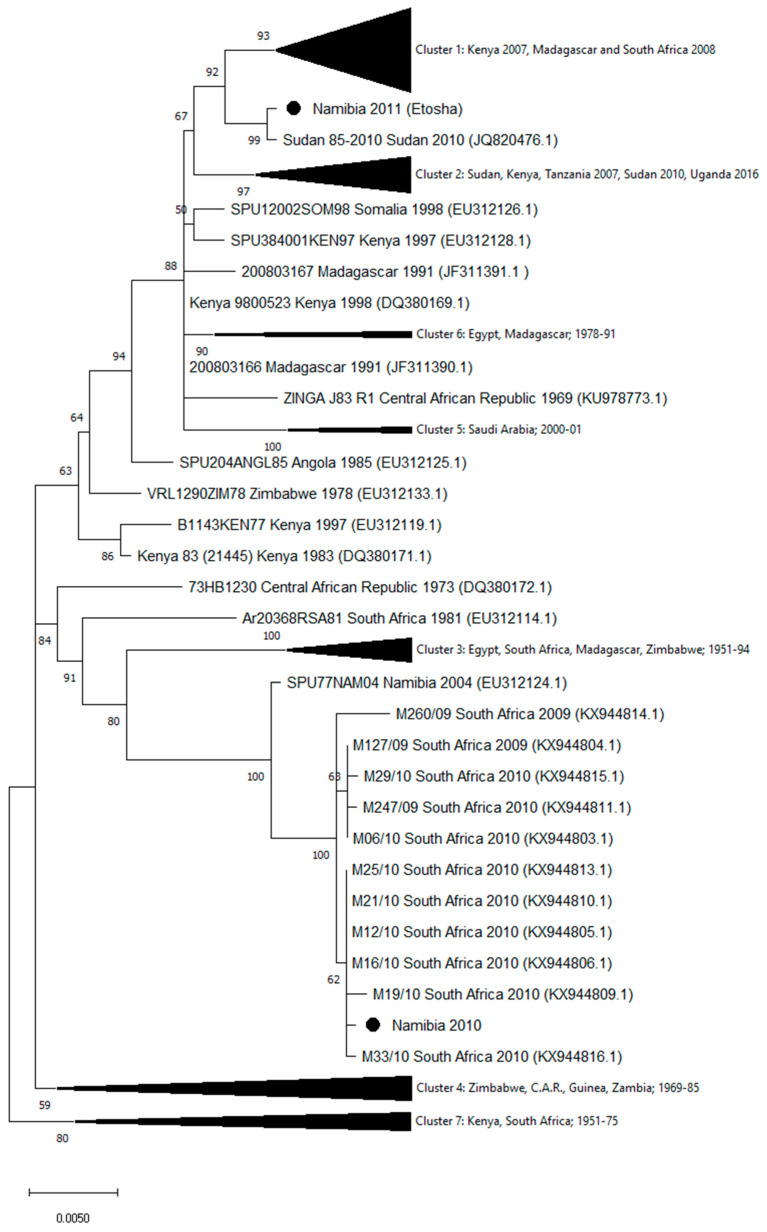
Phylogenetic tree of RVFV generated by the maximum likelihood method with bootstrap (500 replicates) based on 1690 nucleotides of the S genomic segment of RVFV. Analysis involved 125 sequences available in GenBank. Compressed Cluster 1 includes 41 sequences collected in Kenya in 2007 and in Madagascar and South Africa in 2008; Cluster 2: nineteen sequences collected in Sudan, Kenya, and Tanzania in 2007, in Sudan in 2010, and in Uganda in 2016; Cluster 3: thirteen sequences from Egypt, South Africa, Madagascar, and Zimbabwe, collected from 1951 to 1994; Cluster 4: eleven sequences from Zimbabwe, C.A.R., Guinea, and Zambia, collected between 1969 and 1985; Cluster 5: three sequences from Saudi Arabia (2000–2001); Cluster 6: three sequences from Egypt and Madagascar (1978–1991); Cluster 7: nine sequences from Kenya and South Africa, collected between 1951 and 1975. Strain names and accession numbers of the sequences included in the compressed clusters are listed in Table S4.

The homologous sequences in public databases were identified by the Basic Local Alignment Search Tool (www.ncbi.nlm.nih.gov). Sequences were aligned by using MUSCLE (multiple sequence comparison by log-expectation) software [25] available in MEGA version X [26]. Phylogenetic relationships of the Namibia 2010 and Namibia 2011 strains were analyzed using the entire sequences of the L, M, and S genomic segments from all homologous sequences available in GenBank (as of March 2020). Phylogenetic trees were generated using the maximum likelihood method with bootstrap (500 replicates) in MEGA version X (Figure 1, Figure 2 and Figure 3). Tables S1–S3 report the names and the GenBank accession numbers of the sequences included in the compressed clusters.

## 3. Results

Genomic sequences of strains isolated in Namibia during the 2010 and 2011 outbreaks showed a high number of mismatches. In particular, 221 nucleotide differences in the L segment, 134 in the M, and 46 in the S segments were observed. The phylogenetic analysis of the segment sequences generated in this study and all the available RVFV L, M, and S sequences are displayed in Figure 1, Figure 2 and Figure 3. Trees showed that the 2010 Namibia isolate was embedded in a group of strains, which were isolated in 2009 and 2010 RVFV outbreaks in South Africa livestock, particularly M12/10 (KX944850.1; KX944827.1; KX944805.1), M21/10 (KX944856.1; KX944833.1; KX944810.1), M19/10 (KX944853.1; KX944830.1; KX944809.1), M16/10 (KX944852.1; KX944829.1; KX944806.1), and M33/10 2010 (KX944862.1; KX944839.1; KX944816.1). In comparison, the Namibia 2011 strain collected from a wild springbok in Etosha National Park was unrelated to the southern Namibia and South African strains of 2010. The 2011 Namibia strain was genetically close to strains 85-2010 (JQ820485; JQ820488.1; JQ820476.1) and Sudan 7-2010 (JQ820487.1), which were isolated in Sudan, Gezira State, in October 2010 from human cases [27]. Phylogenetic relationships of Namibia 2010 and Namibia 2011 isolates in relation to other isolates available in GenBank, using maximum likelihood trees, showed general coherence among the tree topologies of the individual genome segments (Figure 1, Figure 2 and Figure 3), suggesting that recombination and re-assortment events did not occurred.

## 4. Discussion

Phylogeny based on the entire genome confirms the isolate Namibia 2010 belongs to a single genetic group, together with RVFV strains collected in South Africa in 2009 and 2010. In February 2010, a large epidemic of RVF started across most of the provinces of South Africa and continued until June 2010; 484 outbreaks were reported with 14,342 animal cases in total, which were mostly sheep followed by cattle and goats, and 8877 animal deaths [11]. During 9 May–30 July 2010, the presence of RVFV was confirmed by laboratory tests in seven sheep holdings in Hardap and Karas regions [13]. Previous findings from our group’s molecular characterization, based on the analysis of partial sequences of the M segment of RVFV strains, suggested that Namibian outbreaks were probably caused by RVFV strains originating from South Africa [13].

The observation of genome segment combinations from viruses with different geographic origins strongly suggest that a re-assortment event can occur during the coinfection of two or more virus strains in a single host. Evidence of genome segment re-assortment was described by Sall et al. [17] and by Bird et al. [28]. Grobbelaar and co-workers [14] reported the detection of a re-assorted RVFV from a patient in South Africa potentially exposed to co-infection with live animal vaccine and wild virus. In our study, we found no evidence of genetic re-assortment in the two different RVFV isolates from Namibia.

Molecular data issued from the analysis of wildlife specimens revealed that RVFV strain circulating in northern Namibia in 2011 was close to the RVFV isolates collected from a human population in Sudan in 2010, which belong to the large lineage containing RVFV strains that caused the eastern Africa outbreak in 2006–2008 [24]. Contemporaneous detection of RVFV in domestic livestock and wildlife in the same area may suggest the circulation of a single RVF strain [14]. However, no data are available to determine if these outbreaks were caused by introduced or circulating strains in this endemic area. No genetic relationship was found relative to the earlier Namibia 2010 strain. Results from the current analysis of the Namibian 2011 strain sequences show that closely-related strains can be isolate at large distances, supporting the hypothesis that RVFV can move across large regions of Africa. Sequences from Namibia suggest that the strains collected in 2010 and in 2011 had different origins. This picture shows similarities with what was previously observed by Soumaré et al. (2012) in Mauritania and Senegal [29]. Result from this study support the importance of improving risk-based surveillance and monitoring of RVF to better control the disease through the more efficient use of the prediction tools and of the preventive measures, such as vector control, control of livestock movement, and vaccination. 

The ability to generate full length sequences of viral genomes allows virus variation between epizootic events to be monitored and rapid progress to be made in understanding the epidemiology of RVF. Implementing surveillance in wild animals, humans, and livestock together with the use of forecasting tools now available is crucial to enable rapid responses to the disease and prevention of future widespread epidemics, thus providing health authorities an opportunity to anticipate and prepare for RVF outbreaks.

## Supplementary Materials

The following are available online at https://www.mdpi.com/1999-4915/12/12/1453/s1, Figure S1 Areas of circulation of the viral strains Namiba_2010 (southern Namibia) and Namibia 2011 (northern Namibia), Table S1_Primers used for amplification and sequencing of the entire genome of Namibia_2010 RFV isolate, Table S2_ Segment L tree_clusters, Table S3_ Segment M tree_clusters, Table S4_ Segment S tree_clusters.
