# Supplementary material for: Genetic Diversity of Rift Valley Fever Strains Circulating in Namibia in 2010 and 2011
Source: Viruses. 2020 Dec 16;12(12):1453. doi: 10.3390/v12121453 (PMC7765780; doi:10.3390/v12121453)
Supplement: Supplementary file 1 [file viruses-12-01453-s001.zip › Table S.1.docx]

Table S.4

Primers used for amplification and sequencing of the entire RNA genome of Namibia_2010 RFV isolate from Namibia, 2010

| **Amplification and sequencing of S segment (1690bp)** |
| --- |
|  |
| RVF_S_Fwd_New: 5’-ACACAAAGACCCCCTAGTGCTTAT C -3’ |
| RVF_S_751R: 5’-AATCCAGTTGTTTCTCCCCATCA-3’ |
|  |
| RVF_S_667F: 5’-AAACTCTGGCCTCATTGCTCTTAT-3’ |
| RVF_S_1324R: 5’-CCCTGGCTGGCTGGACAT-3’ |
|  |
| RVF_S_859F: 5’-CCCCAATCCCGACCGTAACC-3’ |
| RVF_S_Rev_new: 5’-acacaaagctccctagagatac-3’ |
|  |
| **Amplification and sequencing of M segment (3885bp)** |
|  |
| RVF_M-AFwd: 5’-ACACAAAGACGGTGC-3’ |
| RVF_M_518R: 5’-GTGCCCCTTCCCTGGTCTGT-3’ |
|  |
| RVF_M_427F:5’-TGACAGTCCTTCCAGCCTTAGCAG-3’ |
| RVF_M_990R:5’-CTTCGCAGACCCCTTTCATTTTTG-3’ |
|  |
| RVF_M_821F:5’-TTCAGTCAAGTGCCCTCCTAAG-3’ |
| RVF_M_1356R:5’-GTATCTGCACAATCCCTGACC-3’ |
|  |
| RVF_M_1262F:5’-TGGGGACGCAGCATTTTG-3’ |
| RVF_M_1713R:5’-GCACTAAGCACGGGTCCTG-3’ |
|  |
| RVF_M_1629F:5’-ATAGGGGTTCACATGGCACACGA-3’ |
| RVF_M_2231R:5’-GACCCCCTTCAACATCAAACAA-3’ |
|  |
| RVF_M_2105F:5’-TCAGGCAAGCTCCAGAATC-3’ |
| RVF_M_2702R:5’-TGCGTCCAGTGAGAGGCTAAC-3’ |
|  |
| RVF_M_2577F:5’-ATCGACTGGGTGCATAAACTCA-3’ |
| RVF_M_3107R:5’-ACAAGATACGGCTGCTCCCACAAA-3’ |
|  |
| RVF_M_2866F:5’-GGGCACCAAACCTTATCTCAT-3’ |
| RVF_M_3601R:5’-TTAGTAGCAGCAAGCCACATTTT-3’ |
|  |
| RVF_M_2866F: 5’-GGGCACCAAACCTTATCTCAT-3’ |
| RVFM-ARev: 5’-ACACAAAGACCGGTGC-3’ |
|  |
| **Amplification and sequencing of L segment (6404bp)** |
|  |
| RVFL-AFwd: 5’-ACACAAAGGCGCCCAATC-3’ |
| RVF_L_520R: 5’-AGGCGCTAACAACATAAAGAACA-3’ |
|  |
| RVF_L_429F:5’-CCAGGCTGCCATGACTAAACTC-3’ |
| RVF_L_928R:5’-TCTCTATTGCCAAGGAACACTCT-3’ |
|  |
| RVF_L_860F:5’-TTGCTGAAGGGAATGATAAAGTT-3’ |
| RVF_L_1493R:5’-TCTGATAAGGATGGCTGATGAAT-3’ |
|  |
| RVF_L_1401F:GTGGGCAACAGATGAGGAC-3’ |
| RVF_L_1993R:CCAAAAGGGTTAATAAAGAAAGTT-3’ |
|  |
| RVF_L_1875F:5’-CTGGGCAGAAGCATTTG-3’ |
| RVF_L_2397R:5’-GAAGGCATCTGACTGTGGT-3’ |
|  |
| RVF_L_2122F:5’-GTGGCATGTTTAATCCTTTTTC-3’ |
| RVF_L_2635R:5’-ATCTGCCTCATACAATCCTCAA-3’ |
|  |
| RVF_L_2514F:5’-GGAGCAAATAGACAACCAGA-3’ |
| RVF_L_3071R:5’-GGCCACCATTTAGGAGAG-3’ |
|  |
| RVF_L_2900F:5’-CCCCCAATAAAGTGAAAAT-3’ |
| RVF_L_3546R:5’-CTCTGAGGGGTAAATGGCAAGGTA-3’ |
|  |
| RVF_L_3438F:5’-CTTCCCAGCTGATGATGAGA-3’ |
| RVF_L_3994R:5’-TAGCAGCTGAGCCCTTGACCTTC-3’ |
|  |
| RVF_L_3890F:5’-GCGGGCTGGGAGGTTTTA-3’ |
| RVF_L_4392R:5’-TGCAGCCATCTTTTGTAGC-3’ |
|  |
| RVF_L_4223F:5’-AAGTCCATAGCCCAGGTGTTGTG-3’ |
| RVF_L_4814R:5’-GGGGCTCCTGTTATTCTGACTGTT-3’ |
|  |
| RVF_L_4630F:5’-AAGAGTAGGATAGGCAAAACAACC-3’ |
| RVF_L_5143R:5’-TTCCGGCTCCCATCTCTTCA-3’ |
|  |
| RVF_L_4902F:5’-CCTTGCTGGCTTCACTCGTA-3’ |
| RVF_L_5420R:5’-CAATATCTGGCCCCTCTGCT-3’ |
|  |
| RVF_L_5360F:5’-ACATGGGGGTCACAAACAAC-3’ |
| RVF_L_5917R:5’-TTAACGCATCATAGTCAACATCAT-3’ |
|  |
| RVF_L_5835F:5’-TTCCCTAAGATCAAAAGTCG-3’ |
| RVF_L-ARev: 5’-ACACAAAGACCGCCCAATATTG-3’ |
