# Supplementary material for: Genetic Diversity of Rift Valley Fever Strains Circulating in Namibia in 2010 and 2011
Source: Viruses. 2020 Dec 16;12(12):1453. doi: 10.3390/v12121453 (PMC7765780; doi:10.3390/v12121453)
Supplement: Supplementary file 1 [file viruses-12-01453-s001.zip › Table S2_ Segment L tree_clusters.docx]

Table 1

The following sequences are included in the clusters indicated in figure 1

Cluster 1: Kenya, Madagascar, Mayotte, South Africa, Tanzania; 2006-09

| 200803170 Madagascar 2008 (JF311376.1) |
| --- |
| 200803165 Madagascar 2008 (JF311371.1) |
| 200803163 Madagascar 2008 (JF311369.1) |
| 2007002060 Madagascar 2008 (EU574013.1) |
| 2008/00099 Mayotte 2008 (HE687304.1) |
| 2007002820 Kenya 2007 (EU574008.1) |
| 2007002445 Kenya 2007 (EU574011.1) |
| 2007000225 Kenya 2007 (EU574025.1) |
| 2007000222 Kenya 2007 (EU574028.1) |
| 2007004193 Kenya 2007 (EU574005.1) |
| 2007003644 Kenya 2007 (EU574006.1) |
| 2007001809 Kenya 2006 (EU574015.1) |
| 2007003081 Kenya 2007 (EU574007.1) |
| 200803162 Madagascar 2008 (JF311368.1) |
| 2007002482 Kenya 2007 (EU574009.1) |
| M47/08 South Africa 2008 (KX944865.1) |
| M80/08/2 South Africa 2008 (KX944869.1) |
| M66/09 South Africa 2009 (KX944868.1) |
| M85/08 South Africa 2008 (KX944871.1) |
| M84/08 South Africa 2008 (KX944870.1) |
| 2007000324 Tanzania 2007 (JF326190.1) |
| M39/08 South Africa 2008 (KX944864.1) |
| TAN/Dod-002/07 Tanzania 2007 (HM586960.1) |
| 200803164 Madagascar 2008 (JF311370.1) |
| M48/08 Madagascar 2008 (KX944866.1) |
| 200803168 Madagascar 2008 (JF311374.1) |
| Kenya-128b-15 Kenya 2006 (KX096938.1) |
| 2007002476 Kenya 2007 (EU574010.1) |
| 2008/00101 Mayotte 2008 (HE687305.1) |
| M37/08 South Africa 2008 (KX944863.1) |
| KEN/Bar-035/07 Kenya 2007 (HM586958.1) |
| KEN/Gar-008/06 Kenya 2006 (HM586954.1) |
| 200803169 Madagascar 2008 (JF311375.1) |
| KEN/Bar-032/07 Kenya 2007 (HM586957.1) |
| 2007000234 Kenya 2007 (JF326186.1) |
| KEN/Kil-006/07 Kenya 2007 (HM586955.1) |
| KEN/KLF-Msq Madagascar 2008 (HM586962.1) |
| KEN/Mal-032/07 Kenya 2007 (HM586956.1) |
| 2007000323 Tanzania 2007 (JF326189.1) |
| 2007001107 Kenya 2007 (EU574020.1) |
| KEN/Gar-Msq Kenya 2006 (HM586961.1) |
| 2007001292 Kenya 2007 (EU574019.1) |
| KEN/Gar-004/06 Kenya 2006 (HM586953.1) |

Cluster 2: Kenya, Sudan, Tanzania, Uganda; 2006-16

| Sudan 2V-2007 Sudan 2007 (JQ820483.1) |
| --- |
| 201601292 Uganda 2016 (MG953422.1) |
| 201601502 Uganda 2016 (MG953421.1) |
| Sudan 86-2010 Sudan 2010 (JQ820484.1) |
| Sudan 28-2010 Sudan 2010 (JQ820486.1) |
| 2007001811 Kenya 2006 (EU574014.1) |
| TAN/Tan-001/07 Tanzania 2007 (HM586959.1) |
| 2007001564 Kenya 2007 (EU574017.1) |
| 2007000094 Kenya 2007 (EU574029.1) |
| 2007004194 Kenya 2007 (EU574004.1) |

Cluster 3: Kenya, Saudi Arabia; 1998-2001

| Kenya 9800523 Kenya 1998 (DQ375400.1) |
| --- |
| Saudi 2000-10911 Saudi Arabia 2000 (DQ375401.1) |
| SA01-1322 Saudi Arabia 2001 (KX096941.1) |

Cluster 4: Zimbabwe, Egypt, Madagascar; 1974-79

| 2250/74 Zimbabwe 1974 (DQ375413.1) |
| --- |
| MgH824 Madagascar 1979 (DQ375414.1) |
| T1 Egypt 1977 (DQ375407.1) |
| ZH-501-777 Egypt 1977 (DQ375408.1) |
| T-46 (228113) Egypt 1977 (DQ375405.1) |
| ZS-6365 Egypt 1979 (DQ375410.1) |
| ZH-1776 Egypt 1978 (DQ375411.1) |
| ZM-657 Egypt 1978 (DQ375409.1) |
| ZC-3349 Egypt 1978 (DQ375412.1) |
| ZH-548 Egypt 1977 (DQ375403.1) |
| MP-12 Egypt 1977 (DQ375404.1) |
|  |
|  |
|  |

Cluster 5: C.A.R, Guinea, Zimbabwe; 1969-85

| 74HB59 Central African Republic 1974 (DQ375415.1) |
| --- |
| 1260/78 Zimbabwe 1978 (DQ375418.1) |
| Zinga Central African Republic 1969 (DQ375419.1) |
| ANK-6087 Guinea 1984 (DQ375421.1) |
| 1853/78 Zimbabwe 1978 (DQ375424.1) |
| CAR-R1622 Central African Republic 1985 (DQ375423.1) |
| Hv-B375 Central African Republic 1985 (DQ375422.1) |
