# Supplementary material for: Genetic Diversity of Rift Valley Fever Strains Circulating in Namibia in 2010 and 2011
Source: Viruses. 2020 Dec 16;12(12):1453. doi: 10.3390/v12121453 (PMC7765780; doi:10.3390/v12121453)
Supplement: Supplementary file 1 [file viruses-12-01453-s001.zip › Table S3_Segment M tree_clusters.docx]

Table 2

The following sequences are included in the clusters indicated in figure 2

Cluster 1: Kenya, South Africa, Madagascar, Tanzania; 2006-09

| M47/08 South Africa 2008 (KX944842.1) |
| --- |
| M84/08 South Africa 2008 (KX944847.1) |
| M85/08 South Africa 2008 (KX944848.1) |
| M66/09 South Africa 2009 (KX944845.1) |
| M80/08/2 South Africa 2008 (KX944846.1) |
| 2007001809 Kenya 2006 (EU574041.1) |
| 2007000080 Kenya 2007 (EU574056.1) |
| 2007001602 Kenya 2007 (EU574043.1) |
| 2007002476 Kenya 2007 (EU574036.1) |
| 2007000260 Kenya 2006 (JF326193.1) |
| 200803170 Madagascar 2008 (JF311385.1) |
| 200803165 Madagascar 2008 (JF311380.1) |
| 200803163 Madagascar 2008 (JF311378.1) |
| 2007000324 Tanzania 2007 (JF326195.1) |
| 2007002482 Kenya 2007 (EU574035.1) |
| 2007002445 Kenya 2007 (EU574037.1) |
| TAN/Dod-002/07 Tanzania 2007 (HM586971.1) |
| TAN/Tan-001/07 Tanzania 2007 (HM586970.1) |
| KEN/Gar-008/06 Kenya 2006 (HM586965.1) |
| M48/08 Madagascar 2008 (KX944843.1) |
| M48/08 Madagascar 2008 (HQ009512.1) |
| 200803168 Madagascar 2008 (JF311383.1) |
| 200803164 Madagascar 2008 (JF311379.1) |
| 2007004193 Kenya 2007 (EU574032.1) |
| 2007000253 Kenya 2006 (JF326192.1) |
| 2008/00101 Mayotte 2008 (HE687306.1) |
| 2008/00099 Mayotte 2008 (HE687303.1) |
| M37/08 South Africa 2008 (KX944840.1) |
| 2007003081 Kenya 2007 (EU574034.1) |
| M39/08 South Africa 2008 (KX944841.1) |
| 2007002060 Kenya 2007 (EU574039.1) |
| 200803162 Madagascar 2008 (JF311377.1) |
| KEN/Bar-035/07 Kenya 2007 (HM586969.1) |
| KEN/Mal-032/07 Kenya 2007 (HM586967.1) |
| KEN/Bar-032/07 Kenya 2007 (HM586968.1) |
| KEN/Kil-006/07 Kenya 2007 (HM586966.1) |
| KEN/KLF-Msq/091/07 Kenya 2007 (HM586973.1) |
| KEN07-KLF112 Kenya 2007 (EF467177.1) |
| 2007000234 Kenya 2007 (JF326191.1) |
| KEN/Gar-004/06 Kenya 2006 (HM586964.1) |
| 2007000323 Tanzania 2007 (JF326194.1) |
| 2007001107 Kenya 2007 (EU574047.1) |
| KEN/Gar-Msq/131B-04/06 Kenya 2006 (HM586972.1) |
| 2007001292 Kenya 2007 (EU574046.1) |
| 2007000618 Kenya 2007 (EU574042.1) |
| KEN07-KUR340 Kenya 2007 (EF460404.1) |
| 200803169 Madagascar 2008 (JF311384.1) |

Cluster 2: Kenya, Sudan, Uganda; 2006-16

| Sudan 2V-2007 Sudan 2007 (JQ820490.1) |
| --- |
| 201601292 Uganda 2016 (MG953419.1) |
| 201601502 Uganda 2016 (MG953420.1) |
| Sudan 86-2010 Sudan 2010 (JQ820489.1) |
| Sudan 28-2010 Sudan 2010 (JQ820491.1) |
| 2007001811 Kenya 2006 (EU574040.1) |
| KEN06-ELH131B08 Kenya 2006 (EF467178.1) |
| 2007000094 Kenya 2007 (EU574055.1) |
| 2007004194 Kenya 2007 (EU574031.1) |
| 2007001564 Kenya 2007 (EU574044.1) |

Cluster 3: Kenya, Saudi Arabia; 1998-2001

| Kenya 9800523 Kenya 1998 (DQ380196.1) |
| --- |
| Saudi 2000-10911 Saudi Arabia 2000 (DQ380197.1) |
| SA01-1322 Saudi Arabia 2001 (KX096942.1 ) |

Cluster 4: C.A.R., Zimbabwe, Guinea; 1969-85

| ANK-3837 Guinea 1981 (DQ380215.1) |
| --- |
| ANK-6087 Guinea 1984 (DQ380216.1) |
| Zinga Central African Republic 1969 (DQ380217.1) |
| 1853/78 Zimbabwe 1978 (DQ380220.1) |
| CAR-R1622 Central African Republic 1985 (DQ380219.1) |
| Hv-B375 Central African Republic 1985 (DQ380218.1) |

Cluster 5: Zimbabwe, Egypt, Madagascar; 1974-79

| 2250/74 Zimbabwe 1974 (DQ380209.1) |
| --- |
| MgH824 Madagascar 1979 (DQ380210.1) |
| T-46 (228113) Egypt 1977 (DQ380199.1) |
| ZH-501-777 Egypt 1977 (DQ380202.1) |
| T1 Egypt 1977 (DQ380201.1) |
| ZH-501 Egypt 1977 (DQ380200.1) |
| ZH-1776 Egypt 1978 (DQ380203.1) |
| ZS-6365 Egypt 1979 (DQ380205.1) |
| ZM-657 Egypt 1978 (DQ380204.1) |
| ZC-3349 Egypt 1978 (DQ380207.1) |
| ZH-548 Egypt 1977 (DQ380206.1) |
| MP-12 (DQ380208.1) |
| Vaccine strain ZH-548M12 (M25276.1) |
