# Supplementary material for: Genetic Diversity of Rift Valley Fever Strains Circulating in Namibia in 2010 and 2011
Source: Viruses. 2020 Dec 16;12(12):1453. doi: 10.3390/v12121453 (PMC7765780; doi:10.3390/v12121453)
Supplement: Supplementary file 1 [file viruses-12-01453-s001.zip › Table S4_Segment S tree_clusters.docx]

Table 3

The following sequences are included in the clusters indicated in figure 3

Cluster 1: Kenya 2007, Madagascar and South Africa 2008

| **Strain name Origin Year (sequence ID)** |
| --- |
| 2007000253 Kenya 2006 (JF326201.1) |
| 00245 Kenya 2006 (JF326199.1) |
| TAN/Dod-002/07 Tanzania 2007 (HM586982.1 ) |
| 2007001800 Kenya 2007 (EU574070.1) |
| 2007000080 Kenya 2007 (EU574087.1) |
| 008/00099 Mayotte 2008 (HE687302.1) |
| 2008/00101 Mayotte 2008 (HE687307.1) |
| 2007002820 Kenya 2007 (EU574061.1) |
| 2007000003 Kenya 2007 (JF326196.1) |
| KEN/KLF-Msq091/07 Kenya 2007 (HM586984.1) |
| 2007001602 Kenya 2007 (EU574071.1) |
| 200803162 Madagascar 2008 (JF311386.1) |
| SPU10307KEN07 Kenya 2007 (EU312146.1) |
| SPU77RSA08 South Africa 2008 (EU709747.1) |
| M37/08 South Africa 2008 (KX944817.1) |
| 2007003644 Kenya 2007 (EU574059.1) |
| SPU2223KEN07 Kenya 2007 (EU312140.1) |
| KEN/Bar-035/07 Kenya 2007 (HM586980.1) |
| 2007000234 Kenya 2007 (JF326198.1) |
| 2007000226 Kenya 2007 (EU574081.1) |
| 2007000222 Kenya 2007 (EU574085.1) |
| M85/08 South Africa 2008 (KX944825.1) |
| M84/08 South Africa 2008 (KX944824.1) |
| M80/08/2 South Africa 2008 (KX944823.1) |
| M47/08 South Africa 2008 (KX944819.1) |
| M39/08 South Africa 2008 (KX944818.1) |
| 2007000250 Kenya 2006 (JF326200.1) |
| KEN/Mal-032/07 Kenya 2007 (HM586978.1) |
| KEN/Bar-032/07 Kenya 2007 (HM586979.1) |
| 200803169 Madagascar 2008 (JF311393.1) |
| 200803164 Madagascar 2007 (JF311388.1) |
| M48/08 Madagascar 2008 (KX944820.1) |
| 200803168 Madagascar 2008 (JF311392.1) |
| 2007004193 Kenya 2007 (EU574058.1) |
| 2007002060 Kenya 2007 (EU574066.1) |
| 2007001292 Kenya 2007 (EU574074.1) |
| 2007000323 Tanzania 2007 (JF326203.1) |
| KEN/Gar-Msq131B-04/06 Kenya 2006 (HM586983.1) |
| KEN/Gar-008/06 Kenya 2006 (HM586976.1) |
| KEN/Gar-004/06 Kenya 2006 (HM586975.1) |

Cluster 2: Sudan, Kenya, Tanzania 2007, Sudan 2010, Uganda 2016

| 201601292 Uganda 2016 (MG953425.1) |
| --- |
| 201601502 Uganda 2016 (MG953426.1) |
| Sudan 86-2010 Sudan 2010 (JQ820477.1) |
| Sudan 2V-2007 Sudan 2007 (JQ820472.1) |
| SPU2214KEN07 Kenya 2007 (EU312141.1) |
| Sudan B-2007 Sudan 2007 (JQ840746.1) |
| Sudan B-2007 Sudan 2007 (JQ840746.1) |
| Sudan 30-2010 Sudan 2010 (JQ820481.1) |
| Sudan 34-2010 Sudan 2010 (JQ820475.1) |
| Sudan 28-2010 Sudan 2010 (JQ820474.1) |
| Sudan 133-2007 Sudan 2007 (JQ820478.1) |
| Sudan 77-2007 Sudan 2007 (JQ820482.1) |
| Sudan 4-2010 Sudan 2010 (JQ820473.1) |
| R2007002059 Kenya 2007 (EU574067.1) |
| SPU2201KEN07 Kenya 2007 (EU312139.1) |
| 2007000665 Kenya 2007 (EU574076.1) |
| TAN/Tan-001/07 Tanzania 2007 (HM586981.1) |
| 2007004194 Kenya 2007 (EU574057.1) |
| 2007001564 Kenya 2007 (EU574072.1) |

Cluster 3: Egypt, South Africa, Madagascar, Zimbabwe; 1951-94

| T-46 (228113) Egypt (DQ380147.1) |
| --- |
| AnTAMBUL Egypt 1994 (EU312110.1) |
| Vaccine strain Uganda (EU312129.1) |
| T1 Egypt (DQ380150.1) |
| ZH-548 Egypt 1977 (DQ380151.1) |
| MP-12 Egypt 1977 (DQ380154.1) |
| ZC-3349 Egypt 1978 (DQ380152.1) |
| ZS-6365 Egypt 1979 (DQ380145.1) |
| SA-51 (Van Wyck) South Africa 1951 (DQ380158.1) |
| MgH824 Madagascar 1979 (DQ380144.1) |
| 2250/74 Zimbabwe 1974 (DQ380143.1) |
| MgH824 Madagascar 1979 (DQ380144.1) |
| 2250/74 Zimbabwe 1974 (DQ380143.1) |

Cluster 4: Zimbabwe, C.A.R., Guinea, Zambia; 1969-85

| VRL1032ZIM78 Zimbabwe 1978 (EU312132.1) |
| --- |
| 1853/78 Zimbabwe 1978 (DQ380168.1) |
| Hv-B375 Central African Republic 1985 (DQ380161.1) |
| CAR-R1622 Central African Republic 1985 (DQ380160.1) |
| Zinga Central African Republic 1969 (DQ380167.1) |
| ANK-3837 Guinea 1981 (DQ380165.1) |
| SPU45ZAMB85 Zambia 1985 (EU312123.1) |
| 73HB1449 Central African Republic 1973 (DQ380162.1) |
| VRL1887ZIM78 Zimbabwe 1978 (EU312135.1) |
| 1260/78 Zimbabwe 1978 (DQ380164.1) |
| VRL2230ZIM78 Zimbabwe 1978 (EU312136.1) |

Cluster 5: Saudi Arabia; 2000-01

| Ar21229SA00 Saudi Arabia 2000 (EU312115.1) |
| --- |
| SA01-1322 Saudi Arabia 2001 (KX096943.1) |
| Saudi 2000-10911 Saudi Arabia 2000 (DQ380170.1) |

Cluster 6: Egypt, Madagascar; 1978-91

| An999MAD91 Madagascar 1991 (EU312107.1) |
| --- |
| ZH-1776 Egypt 1978 (DQ380153.1) |
| An1000MAD91 Madagascar 1991 (EU312108.1) |

Cluster 7: Kenya, South Africa; 1951-75

| Ar74RSA55 South Africa 1955 (EU312111.1) |
| --- |
| B314KEN62 Kenya 1962 (EU312118.1) |
| Kenya 57 (Rintoul) Kenya 1951 (DQ380155.1) |
| 56KEN65 Kenya 1965 (EU312103.1) |
| Kenya 56 (IB8) Kenya collected pre-1965 (DQ380176.1) |
| H1825RSA75 South Africa 1975 (EU312120.1) |
| Ar12568RSA71 South Africa 1971 (EU312113.1) |
| 35/74 South Africa 1974 (JF784388.1) |
| SA-75 South Africa 1975 (DQ380175.1) |
