# Supplementary figures and images for: Genetic Diversity of Rift Valley Fever Strains Circulating in Namibia in 2010 and 2011
Source: Viruses. 2020 Dec 16;12(12):1453. doi: 10.3390/v12121453 (PMC7765780; doi:10.3390/v12121453)

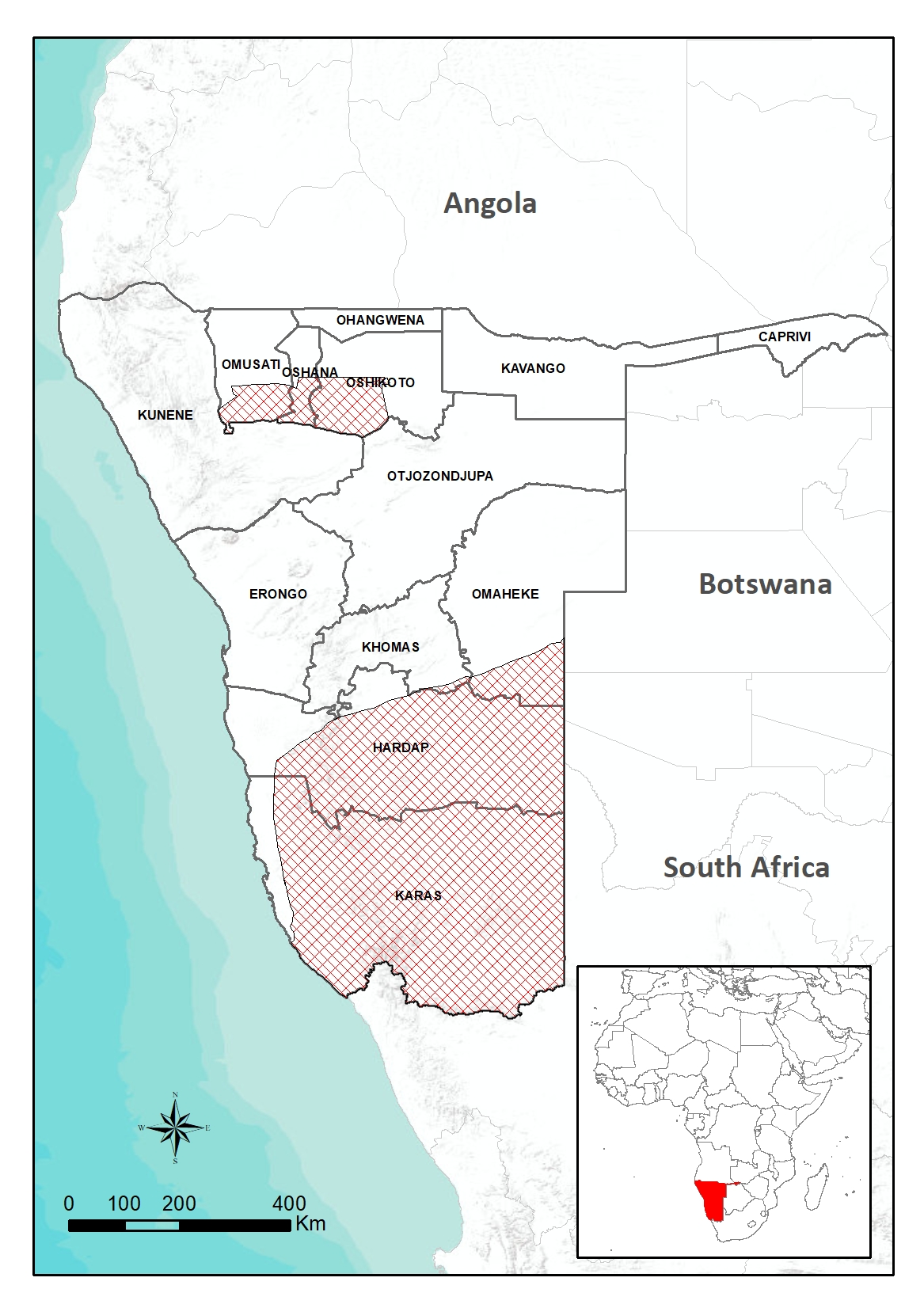

Supplement: Supplementary file 1 [file viruses-12-01453-s001.zip › Figure S1.jpg]
